# Insectivorous Bats Digest Chitin in the Stomach Using Acidic Mammalian Chitinase

**DOI:** 10.1371/journal.pone.0072770

**Published:** 2013-09-03

**Authors:** Sara Strobel, Anna Roswag, Nina I. Becker, Tina E. Trenczek, Jorge A. Encarnação

**Affiliations:** 1 Mammalian Ecology Group, Department of Animal Ecology and Systematics, Justus-Liebig-University of Giessen, Giessen, Germany; 2 Department of General Zoology and Developmental Biology, Justus-Liebig-University of Giessen, Giessen, Germany; National Institute of Agronomic Research, France

## Abstract

The gastrointestinal tract of animals is adapted to their primary source of food to optimize resource use and energy intake. Temperate bat species mainly feed on arthropods. These contain the energy-rich carbohydrate chitin, which is indigestible for the endogenous enzymes of a typical mammalian gastrointestinal tract. However, the gastrointestinal tract of bat species should be adapted to their diet and be able to digest chitin. We hypothesized that (i) European vespertilionid bat species have the digestive enzyme chitinase and that (ii) the chitinolytic activity is located in the intestine, as has been found for North American bat species. The gastrointestinal tracts of seven bat species (*Pipistrellus pipistrellus*, *Plecotus auritus*, *Myotis bechsteinii*, *Myotis nattereri*, *Myotis daubentonii*, *Myotis myotis,* and *Nyctalus leisleri*) were tested for chitinolytic activity by diffusion assay. Gastrointestinal tracts of *P. pipistrellus*, *P. auritus*, *M. nattereri*, *M. myotis,* and *N. leisleri* were examined for acidic mammalian chitinase by western blot analysis. Tissue sections of the gastrointestinal tract of *P. pipistrellus* were immunohistochemically analyzed to locate the acidic mammalian chitinase. Chitinolytic activity was detected in the stomachs of all bat species. Western blot analysis confirmed the acidic mammalian chitinase in stomach samples. Immunohistochemistry of the *P. pipistrellus* gastrointestinal tract indicated that acidic mammalian chitinase is located in the stomach chief cells at the base of the gastric glands. In conclusion, European vespertilionid bat species have acidic mammalian chitinase that is produced in the gastric glands of the stomach. Therefore, the gastrointestinal tracts of insectivorous bat species evolved an enzymatic adaptation to their diet.

## Introduction

Animals have to ingest and digest food to ensure the continuous functioning of their internal metabolism by covering, for example, their energy, protein and vitamin requirements [1]. The multi-stage process of digestion includes mechanical, chemical and enzymatic steps for converting nutrients [2]. Bat species have a high mass-specific energy demand because of their small size and the ability to fly actively [3], [4]. In flying animals, food needs to be processed quickly to reduce the energy demand caused by increased flight mass [2]. European bat species have a diet consisting predominantly of arthropods [5]. They have short retention times [6] but a high digestive efficiency [7]. This suggests that their gastrointestinal (GI) tract is highly adapted to their diet since it digests arthropods quickly and thoroughly. Therefore, it could be argued that European bat species depend on arthropod-specific digestive enzymes. Since arthropods consist of up to 75% chitin (energy content 21.2 kJ/g, [8]), it is highly plausible that bat species are able to digest chitinous material, as has been demonstrated in other vertebrates such as the European green lizard (*Lacerta viridis*), the common blackbird (*Turdus merula*) and the red fox (*Vulpes vulpes*) [9], [10].

Chitin can be degraded by chitinases (EC 3.2.1.14) and some lysozymes (EC 3.2.1.17) [11], [12]. In mammals, only two chitinases have been identified: chitotriosidase and acidic mammalian chitinase (AMCase) [13], both of which are classified as endochitinases [14]. Chitotriosidase is mainly secreted by phagocytes and acts against chitin-containing pathogens [15]. AMCase has so far only been identified in mice (Mus musculus), macaques (Macaca fascicularis) and humans [16], [17]. It is highly expressed in the stomach and lung, indicating a dual digestive and immunological function [16], [17]. Chitinolytic activity can also originate from endogenous enzymes, ingested food present in the GI tract, or enzymes produced by microorganisms [18], [19].

Chitinolytic activity in the GI tract has been found in several insectivorous bat species [8], [9]. However, there is no knowledge about the corresponding enzyme. Jeuniaux [9] verified chitinolytic activity in the GI tract of *Rhinolophus ferrumequinum*, a European bat species of the family Rhinolophidae. Whitaker et al. [8] demonstrated chitinolytic activity in the GI tract of North American vespertilionid bat species of the genera *Myotis*, *Eptesicus*, *Nycticeius*, *Lasiurus*, *Pipistrellus* and *Lasionycteris*. They isolated chitinase-producing bacteria strains from the intestine as a source for the chitinolytic activity. In contrast, Jeuniaux [9] found evidence of chitinolytic activity in the gastric mucosa of the stomach of *Rhinolophus ferrumequinum* whereas the intestine exhibited no chitinolytic activity. However, Buchholz, Wells & Conaway [20] could not detect any chitinase in the insectivorous bat species *Pipistrellus subflavus* and *Myotis grisescens*. Besides chitinases, some lysozymes are able to dissolve chitin [11], [12]. For example, Phillips, Weiss & Tandler [21] detected lysozyme in salivary glands of insectivorous bat species and speculated that it could act as a chitinolytic enzyme in the saliva. However, lysozymes are mainly anti-bacterial and are an important part of the immune system [22] or for digestion of bacteria in ruminants [12].

We hypothesize that (i) European insectivorous bat species of the family Vespertilionidae possess chitinolytic activity in the GI tract, as has been demonstrated for North American insectivorous bat species [8] and one European bat species of the family Rhinolophidae [9] and (ii) the chitinolytic activity is located in the intestine, as has been shown in North American species [8]. In this study, we located chitinolytic activity and identified the corresponding enzyme as AMCase using an enzyme assay, immunoblotting and immunohistochemistry.

## Materials and Methods

### Ethics statement

All individuals used in this study died at voluntary rehabilitation centres for bats. They were delivered by volunteers without any kind of refund. According to the German Animal Welfare Act (TSchG §4 (3)) and to the Federal Nature Conservation Act (BNatSchG §45 (4)) no permission is required to work on carcasses. The mouse stomach was a remnant of a study by the Institute of Anatomy and Cell Biology at the Justus-Liebig-University of Giessen which was approved by the regional council (No. V54-19C20/15C Giessen 20/23 400AZ). No animal was killed for the purposes of this study.

### Tissue storage

Carcasses were stored immediately after death at −20°C. Bats were delivered on ice i.e. frozen to the University of Giessen. The carcasses were stored for a maximum of six months at −80°C until tissue preparation. Macro- and microscopic observations verified the very good preservation of organs and cells that made enzymatic and histological examinations of the tissues possible.

### Tissue preparation

Carcasses of seven insectivorous bat species without any signs of putrefaction (*Pipistrellus pipistrellus* (*n* = 14), *Plecotus auritus* (*n* = 3), *Myotis bechsteinii* (*n* = 1), *Myotis nattereri* (*n* = 3), *Myotis daubentonii* (*n* = 2), *Myotis myotis* (*n* = 1) and *Nyctalus leisleri* (*n* = 1)) were used in this study (Table 1). After opening the abdominal wall, the GI tract was removed, washed with 0.9% NaCl, and dried on filter paper. The GI tract was divided into the esophagus, stomach, duodenum, jejunum/ileum, ileum/colon and colon/rectum after Ishikawa et al. [23] and weighed on a digital scale (EW2200-2NM, accuracy: 0.01 g; Kern & Sohn GmbH, Balingen, Germany). In addition, the stomach of a *Mus musculus* (strain C57BL/6, Black 6; *n* = 1) was used as a positive control for AMCase detection by western blotting.

**Table 1:** Distribution [40], main prey items [5], [41] and IUCN^1^ category [40] of studied bat species.

Species	Distribution^2^	Diet	IUCN category^3^
***Pipistrellus pipistrellus***	Europe, NW Africa, Central Asia	Diptera	LC
***Plecotus auritus***	Europe	Lepidoptera	LC
***Myotis bechsteinii***	Europe, SW Asia	Lepidoptera, Diptera	NT
***Myotis nattereri***	Europe, NW Africa	Diptera, Arachnida	LC
***Myotis daubentonii***	Europe, N Asia, Korea, Japan	Diptera	LC
***Myotis myotis***	Europe	Coleoptera	LC
***Nyctalus leisleri***	Europe, NW Africa	Diptera, Lepidoptera	LC

1 IUCN  =  International Union for Conservation of Nature.

2 NW  =  north-west, SW  =  south-west, N  =  north.

3 LC  =  least concern, NT  =  near threatened.

### Preparation of soluble protein fractions

GI tract segments of non-fixed, fresh specimens of *P. pipistrellus* (*n* = 11), *P. auritus* (*n* = 3), *M. bechsteinii* (*n* = 1), *M. nattereri* (*n* = 3), *M. daubentonii* (*n* = 2), *M. myotis* (*n* = 1) and *N. leisleri* (*n* = 1) and the stomach of *M. musculus* were individually ground up in a mortar and pestle with extra-pure sea sand (Merck, Germany) and 0.9% NaCl (standardized tissue amount: 1 mL per 100 mg tissue). The homogenates were incubated overnight at 4°C [10] and then centrifuged (20 min, 3500 g, 4°C). The supernatants were kept at −20°C until further analysis.

### Determination of chitinolytic activity

To measure chitinolytic activity, agarose gel plates were prepared as described by Zou, Nonogaki & Welbaum [24] with some modifications. Phosphoric acid swollen chitin was prepared by mixing 10 g chitin from crab shells (Roth, Germany) with 100 mL 85% phosphoric acid and incubated for 48 h at 4°C. Then 2 L cold tap water was added and the resulting cake was washed until pH 6.5 was reached [25], [26]. Agarose (1.6%) was dissolved in incubation buffer (pH 5.0) [24] in a microwave oven and cooled to 50–60°C. Afterwards, the phosphoric acid swollen chitin (0.5%) was added and 10 mL of this suspension was pipetted into 85–mm Petri dishes. After polymerization, 4–mm–diameter wells were punched into the agarose and gel pieces were removed using a water-jet pump.

Lyophilized powder of standard chitinase from *Serratia marcescens* (5 U; Sigma-Aldrich, Germany) was dissolved in 1 mL incubation buffer as the standard stock solution. A known concentration of standard chitinase was added to each plate as reference and incubation buffer was used as the negative control. First, 6 µL samples of each solution were pipetted per well, after which the plates were incubated for 20 min at room temperature to allow samples to diffuse into the agar. Then an additional L sample was added to each well and plates were incubated at room temperature for 20 min followed by incubation at 37°C for 20 h. Agarose plates were then stained with 0.1% calcofluor (Calcofluor Brightener M2R; Sigma, MO, USA) for 10 min and washed with distilled water for 2 h. Lytic zones were visualized using UV transillumination and then photographed. Diameters of lytic zones were measured using GIMP (version 2.6.11; www.gimp.org). Using a reference dilution series of the chitinase stock solution with incubation buffer enzyme activities were calculated by zone diameter versus logarithm of concentration and variation between plates were adjusted to internal chitinase standards used on each Petri dish.

To analyze enzymatic activity at different pH values, gel plates were prepared as before but with different pH values (pH 4.0, pH 5.0, pH 6.0, pH 7.0 and pH 8.0). Supernatants of the stomach, duodenum, jejunum/ileum, ileum/colon and colon/rectum of one individual of *P. pipistrellus* were used. The lytic zones were visualized using UV transillumination and analyzed as before. In addition, pH values of the GI tract sections of five individuals of *P. pipistrellus* were measured using multicolor-coded pH paper (pH 0.0–6.0: Acilit, accuracy 0.5; pH 6.5–10.0: Special Indicator, accuracy 0.3; Merck).

### Expression of chitinase in the GI tract

#### Western blot analysis

Western blotting was performed to identify and biochemically locate chitinase in the GI tract of European bat species and to exclude chitinolytic activity caused by lysozymes. Supernatants of tissue samples from six bat species (GI tract section samples (stomach, duodenum, jejunum/ileum, ileum/colon and colon/rectum): *P. pipistrellus* (*n* = 2), *P. auritus* (*n* = 2), *M. nattereri* (*n* = 1), *M. myotis* (*n* = 1) and *N. leisleri* (*n* = 1); additional stomach samples: *P. pipistrellus* (*n* = 9), *M. nattereri* (*n* = 1), *M. daubentonii* (*n* = 2)) and the stomach of a *M. musculus* used as a positive control [27] were subjected to sodium dodecyl sulfate-polyacrylamide electrophoresis (SDS-PAGE) (Laemmli [28] modified after Sambrook, Fritsch & Maniatis [29]).

Supernatants of each 750 µg tissue were mixed 1∶1 in 2× SDS gel-loading buffer and heated to 95°C for 3 min. Of each sample, 15 µL was subjected to a 12% resolving gel and 5% stacking gel. Electrophoresis was carried out under reducing conditions at a voltage of 100 V. The separated proteins were electroblotted for 1 h at a constant current of 0.8 mA/cm^2^ on PVDF membranes. The blots were blocked with 5% non-fat dried milk in tris buffered saline (TBS, pH 7.5) containing 0.1% Tween 20 (Roth) for 1 h before incubation with a rabbit polyclonal antibody directed against the N-terminal of acidic chitinase (AVIVA Systems Biology, CA, USA; diluted 1∶1000 in TBS containing 1% BSA) at 4°C overnight. After washing with TBS containing 0.05% Tween 20 and 0.1% BSA, the membranes were incubated for 1 h with alkaline phosphatase-conjugated goat polyclonal antibody to rabbit IgG (H&L) (Roth, Anti Rabbit-AP 4751; diluted 1∶7500 in TBS containing 1% BSA). The blots were washed four times and antibody binding was visualized by incubation with bromochloroindoyl phosphate (Bethesda Research Laboratories, MD, USA) and nitroblue tetrazolium substrate (Biotech Trade & Service GmbH, Germany) according to Harlow and Lane [30].

#### Immunohistochemistry

To localize AMCase on the cellular level, immunohistochemical analysis was performed on GI tract segments of *P. pipistrellus* (*n* = 3). The GI tract parts were fixed in 4% paraformaldehyde in phosphate-buffered saline (pH 7.0) for 24 h before they were washed 4×1 h with TBS. Then the tissue blocks were dehydrated in a graded ethanol series (30%, 50%, 70%, 90%, 100%) and finally embedded in paraffin. The paraffin blocks were cut into sections of 4–9 µm thickness using a sledge microtome (Leitz, Germany) and were dried overnight. To get accessible antigen binding sites, tissue sections were predigested with pepsin (Sigma) after Goto et al. [27]. The sections were washed with 0.01% Tween 20 in TBS. Nonspecific sites were blocked with 5% goat serum (Merck) in 3% BSA (AppliChem, Germany). The sections were exposed to the rabbit polyclonal antibody directed against the N-terminal of acidic chitinase (AVIVA Systems Biology; diluted 1∶200 in TBS containing 1% BSA) in a moist chamber. Unbound antibodies were removed by washing with TBS, before the secondary antibody (ChromeoTM 546, Abcam, UK; diluted 1∶2500 in 0.5% BSA in TBS) was applied. For nuclear counterstaining sections were incubated with 0.05% 4′,6-diamidino-2-phenylindole (DAPI) (AppliChem). Following a final rinsing with TBS, the sections were mounted with 1,4-diazabicyclo[2.2.2]octane solution (DABCO) (Sigma). For control of autofluorescence and binding specificity of the antibodies the sections were processed with fluorescein isothiocyanate (FITC) labeled secondary antibody but without primary antibody. The sections were evaluated using a fluorescence microscope (Olympus BX60 F-3; Olympus Optical Co LTP, Germany).

## Results

### Chitinolytic activity

**Figure 1:**
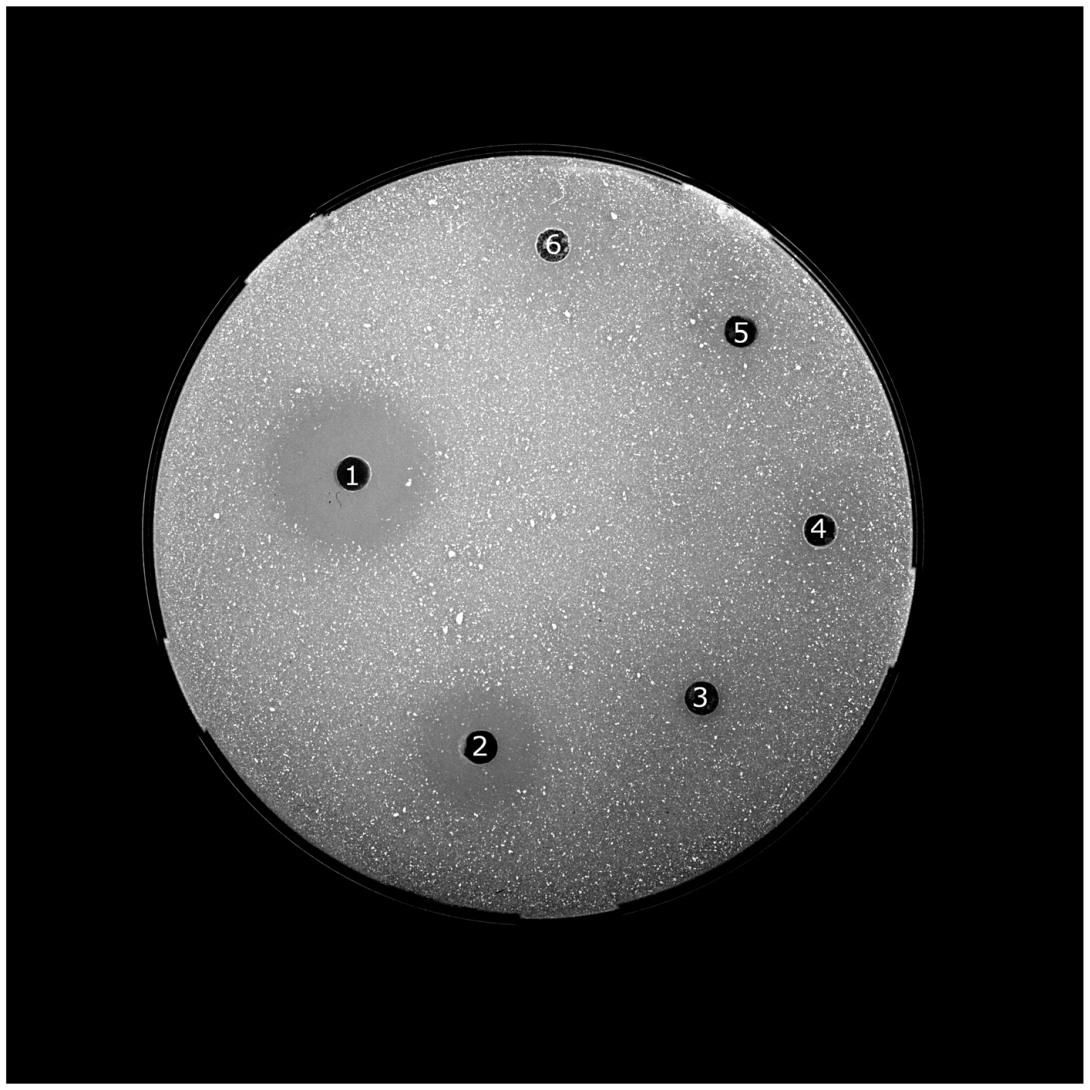
Exemplary gel plate for the measurement of the chitinolytic activity of the GI tract. 1– reference, 2– stomach, 3– duodenum, 4– jejunum/ileum, 5– ileum/colon samples of *Plecotus auritus* and 6– negative control.

**Figure 2:**
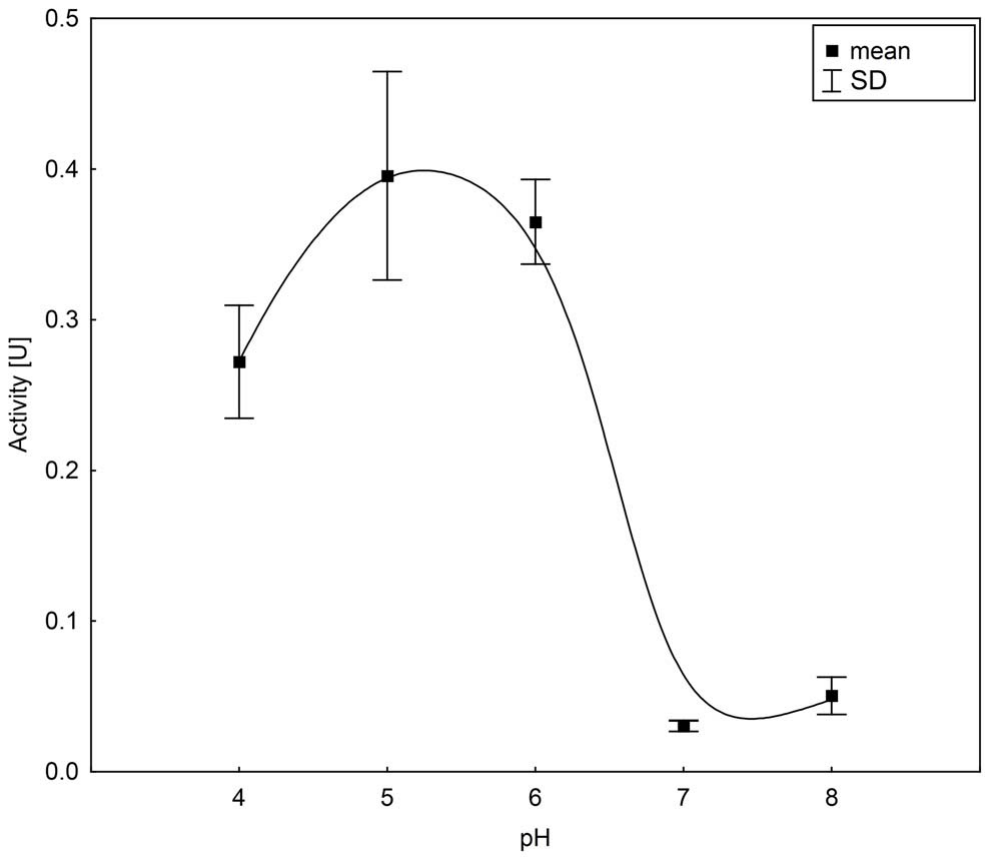
Mean chitinolytic activity in the stomach samples of *Pipistrellus pipistrellus* at different pH values. Curve fitted by distance-weighted least square smoothing procedure.

We were able to detect chitinolytic activity in the stomach samples of all individuals (for example Fig. 1) and in the colon/rectum sample of one, *M. myotis, M. nattereri* and *N. leisleri* each (Table 2). No chitinolytic activity could be measured in the duodenum, jejunum/ileum or ileum/colon samples. The chitinolytic activity in the stomach samples was highest between pH 5.0 and pH 6.0 (Fig. 2). Supporting our previous results, no chitinolytic activity was detected in the other regions of the GI tract, regardless of pH value. The mean pH value of the GI tract of *P. pipistrellus* (*n* = 5) was 5.6±0.2 in the stomach, 7.0±0.3 in the duodenum, 7.1±0.2 in the jejunum/ileum, 7.0±0.2 in the ileum/colon and 7.0±0.5 in the colon/rectum.

**Table 2:** Median chitinolytic activity (min–max) (U) in the GI tract of studied bat species.

Species	GI tract segment	n	Chitinolytic activity (U)
***Pipistrellus pipistrellus***	stomach	13	0.4 (0.1–0.7)
***Plecotus auritus***	stomach	3	0.7 (0.2–0.9)
***Myotis bechsteinii***	stomach	1	0.1
***Myotis nattereri***	stomach	3	0.1 (0.01–0.3)
	colon/rectum	1	0.01
***Myotis daubentonii***	stomach	2	0.1 (0.1–0.2)
***Myotis myotis***	stomach	1	0.3
	colon/rectum	1	0.01
***Nyctalus leisleri***	stomach	1	1.0
	colon/rectum	1	0.1

### Expression of chitinase in the GI tract

**Figure 3:**
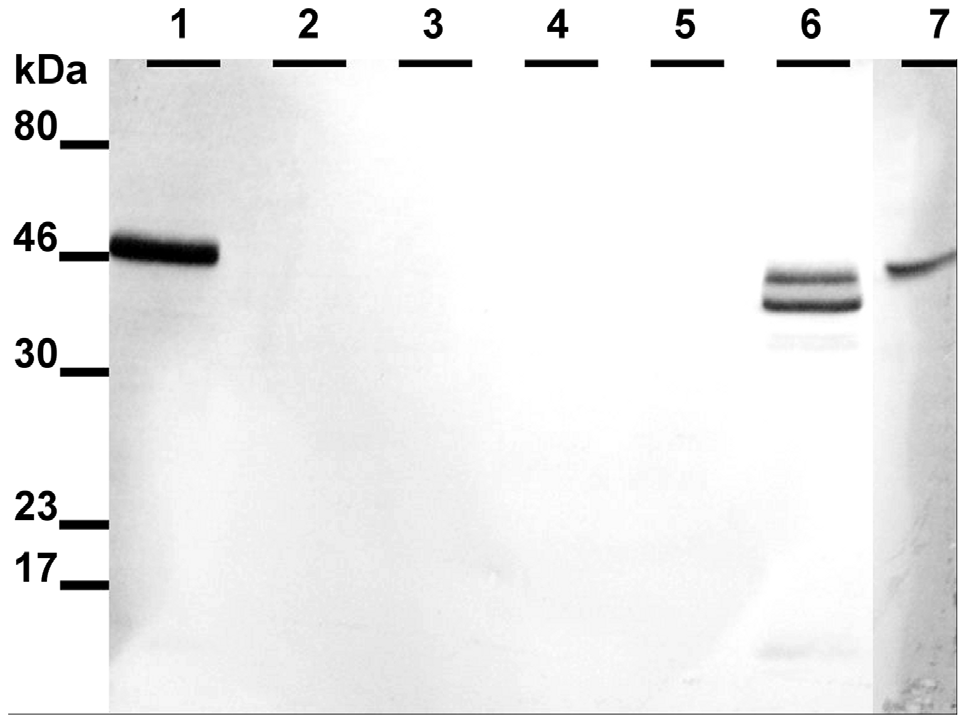
Exemplary western blot analysis of AMCase in the GI tract of *Pipistrellus pipistrellus.* Individual 1: lane 1 =  stomach, lane 2 =  duodenum, lane 3 =  jejunum/ileum, lane 4 =  ileum/colon, lane 5 =  colon/rectum; individual 2: lane 6 =  stomach; lane 7 =  positive control (stomach sample of *Mus musculus*). The AMCase displayed a sharp band in the lane containing stomach proteins (46 k) except that lane 6 contained two distinct bands under 46 k probably caused by proteolytic digestion. Primary antibody dilution 1:1000.

**Figure 4:**
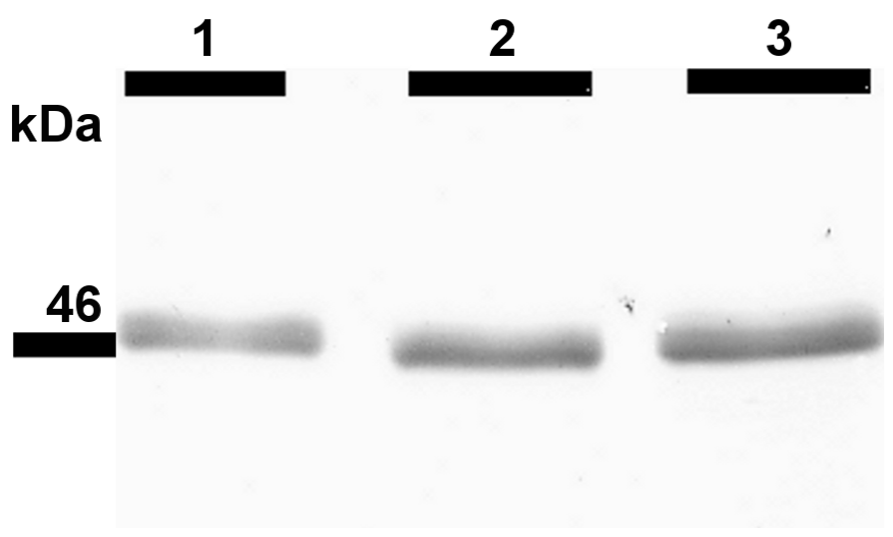
Western blot analysis of AMCase in the stomachs of three different bat species. Lane 1 =  *Plecotus auritus*, lane 2 =  *Myotis myotis*, lane 3 =  *Nyctalus leisleri*. Primary antibody dilution 1∶1000.

**Figure 5:**
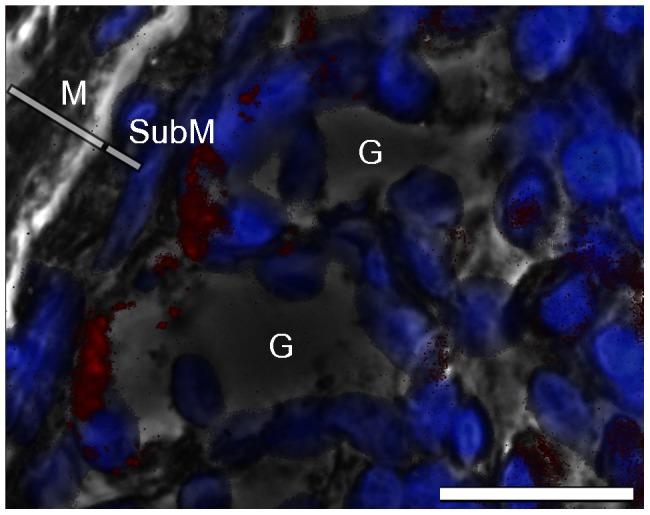
Immunohistochemical analysis of the stomach of *Pipistrellus pipistrellus*. Picture overlay of labeling of the α-AMCase antibody (red); DAPI counterstained (blue) and phase contrast demonstrating positive labeling of the antibody at the bottom of the gastric glands. Bar  = 50 µm. G  =  gastric gland, M  =  mucosa, SubM  =  submucosa.

Western blot analysis of the *M. musculus* stomach showed a characteristic band at a relative molecular weight of 46 k, indicating the presence of AMCase. Furthermore, in all stomach samples of *P. pipistrellus, P. auritus, M. nattereri*, *M. myotis*, and *N. leisleri* a clear protein band at 46 k was identified (for representative western blot images, see Fig. 3 for *Pipistrellus* and Fig. 4 for *Plecotus, Myotis* and *Nyctalus*). This protein band was not detected in the esophagus, duodenum, jejunum/ileum, ileum/colon or colon/rectum samples of the bat species (Fig. 3). All immunohistochemical results were controlled for autofluorescence and unspecific binding of the secondary FITC-coupled antibody. Stomach sections were positive for anti-AMCase antibody labeling, whereas in the esophagus, duodenum, jejunum/ileum, ileum/colon and colon/rectum sections no binding was detected. In the stomach sections, anti-AMCase labeling was limited to the bottom of the gastric glands along the gastric mucosa around the DAPI-stained cell nuclei (Fig. 5).

## Discussion

We hypothesized that European insectivorous bat species of the family Vespertilionidae have the digestive enzyme chitinase. This hypothesis was confirmed by the presence of chitinolytic activity in the stomachs of the studied species. Furthermore, a true chitinase, more particularly AMCase, could be biochemically identified in all stomach samples. Active chitinases are common and conserved among mammals [14]. However, the location and function of the AMCase differ among species and are not completely resolved [31].

We further hypothesized that the chitinolytic activity is located in the intestine, especially in the small intestine, as it is the site where the main enzymatic digestion and absorption takes place [32]. Our results did not confirm this hypothesis as chitinolytic activity was localized mainly in the stomach and for three individuals at low activity levels in the colon/rectum. The high variability of the chitinolytic activity in the studied individuals might be caused by varying digestive activity of individuals at the time of death. This is supported by different amounts of food found in the GI tracts. The chitinolytic activity in stomach samples but not in colon/rectum samples could be traced back to the activity of the AMCase and not to a lysozyme by western-blotting. The activity of bat AMCase was optimal between pH 5.0 and pH 6.0. These pH levels are comparable to the acidic milieu in the stomachs of insectivorous bat species as measured in the present study and reported by Naumova and Zharova [33]. This is a first indication for the biological relevance of AMCase during digestion in this part of the GI tract. However, further experiments like digestive efficiency trials should be conducted to test if the activity of AMCase poses a biological significance to chitin digestion. AMCase has a dual function in immunity and digestion of chitin-containing organisms [34], [35]. For instance, human AMCase is not adapted to the acidic environment in the stomach, unlike the AMCase found in mice [31]. The stomach AMCase of *M. musculus* contains amino acid substitutions that are necessary for the adaption to the acidic milieu of the stomach [31]. Furthermore, Boot et al. [17] demonstrated that the AMCase mRNA of *M. musculus* is only found in the stomach. If these amino acid substitutions are present in the AMCase of bat species remains to be shown.

The immunohistochemical results from this study support the localization of AMCase in the stomach of bat species, particularly in the gastric glands of the mucosa. Furthermore, we found that the enzyme was located in or around the chief cells located at the base of the gastric glands, as was previously shown for the stomach AMCase of *M. musculus* [27], [31], [34]. Chief cells secrete digestive enzymes [36] that are located in the numerous cytoplasmic granules [37]. A common enzyme produced by this gastric cell type is pepsinogen, a precursor of the proteolytic enzyme pepsin [38]. Goto et al. [27] demonstrated that the production site of stomach AMCase of *M. musculus* is in these secretory granules. Therefore, it is most likely that AMCase is also secreted by gastric chief cells in bat species. This is contrary to the results of Whitaker et al. [8], who stated that chitinase in bat species is produced by chitinase-producing bacteria strains (mostly of the family Enterobacteriaceae) in the intestine. It is known that intestinal bacteria produce chitinase to satisfy their own nutritional requirements [39]. However, chitinase-producing enterobacteria can also be found in the GI tracts of mammals that do not feed on chitinous material [19]. This suggests that there is no close connection between chitin digestion and chitinolytic bacteria. In this study, low chitinolytic activity was measured in the intestines of only a few individuals, and no AMCase could be detected when separating the intestine from the stomach. This occasional chitinolytic activity may be explained by transport of the AMCase produced in the stomach into the intestine with the food, as discussed by Suzuki et al. [34] and Boot et al. [17]. Additionally, the low chitinolytic activity in the intestine may be caused by chitinase-producing enterobacteria [8]. However, quantification of these bacteria would be needed to verify the participation in chitin digestion by these symbionts. Therefore, it is plausible that chitin in insectivorous bat species is digested by a combination of endogenous stomach AMCase and chitinase secreted by intestinal bacteria, as was suggested for *M. musculus* [17]. This study clearly demonstrates that European insectivorous bats of the family Vespertilionidae have the digestive enzyme AMCase. We showed that this enzyme is active and located in the stomach, particularly in or around the chief cells at the base of the gastric glands.
